# Peer Review of “Awareness, Experiences, and Attitudes Toward Preprints Among Medical Academics: Convergent Mixed Methods Study”

**DOI:** 10.2196/95737

**Published:** 2026-04-17

**Authors:** Ludo Waltman

**Affiliations:** 1 Leiden University, Leiden, The Netherlands

**Keywords:** preprint, medical academics, publishing attitudes, editorial policies, survey

*This is a peer review report for “Awareness, Experiences, and Attitudes Toward Preprints Among Medical Academics: Convergent Mixed Methods Study.”*

## Round 1 Review

This paper [1] offers valuable insights into attitudes toward preprinting at a medical institution in Istanbul. The research appears sound to me. The paper is clear and easy to read.

I have one important comment: I was unable to find the survey form. I urge the authors to make the survey form, and also the data collected in the survey, openly available. To interpret the results of the survey, it is important to have access to the underlying survey questions.

## Round 2 Review

In their response to my earlier comments and the comments of the other reviewer, the authors refer to supplementary material. I am unable to find this supplementary material. Where can I find it?

Apart from this issue, I am satisfied with the revised article. I have no suggestions for further improvements.
